# Case Report: Fatal massive hemoptysis secondary to pulmonary actinomycosis: a diagnostic and therapeutic challenge in a multidisciplinary approach

**DOI:** 10.3389/fmed.2025.1680917

**Published:** 2025-11-12

**Authors:** Qiong-Fang Yang, Cai-Min Shu

**Affiliations:** Department of Respiratory Medicine, Affiliated Dongyang Hospital of Wenzhou Medical University, Dongyang, Zhejiang, China

**Keywords:** fatal massive hemoptysis, secondary, pulmonary actinomycosis, surgical resection, case report

## Abstract

**Background:**

*Actinomyces* infections tend to involve the head and neck, while lung infections are relatively rare, and fatal hemoptysis due to pulmonary actinomycosis is even rarer.

**Case presentation:**

A 74-year-old male patient who presented with a cough with intermittent hemoptysis for 17 months was admitted to the hospital for the fourth time on June 24, 2024. At the first visit (15 months ago), a computed tomography (CT) scan of the chest revealed a high-density shadow in the right middle bronchus (suggesting a foreign body to be drained) and an infected lesion in the lower lobe of the right lung. Hemoptysis was temporarily relieved after bronchoscopic removal of the foreign body and anti-infective treatment. Four months later, the patient was readmitted to the hospital due to a recurrence of hemoptysis. CT showed a lesion in the right lower lobe of the lung with cavitation, and bronchoscopy showed no abnormality. The pathology of percutaneous lung puncture biopsy suggested acute and chronic inflammation, and the patient was discharged under oral treatment with moxifloxacin. Four months later, the patient was admitted to the hospital for the third time because the hemoptysis volume had increased to 50 ml/day. Digital subtraction angiography-guided bronchial artery embolization was performed, and hemoptysis was controlled after the operation. Six months later, the patient was re-admitted to the hospital because of sudden hemoptysis as an emergency. Various medications were ineffective in stopping the hemoptysis, and the hemoptysis recurred; the volume was about 150 ml, with a sudden drop in blood oxygen saturation level to 82%. Due to the possibility of asphyxiation by blood clot, emergency bronchoscopic balloon occlusion was performed to stop bleeding, and right lower lobectomy was performed after multidisciplinary consultation. Post-operative pathology showed that the lung tissue was accompanied by tracheal dilatation, pus accumulation, and inflammatory granulomatous changes, with positive periodic acid Schiff staining, supporting actinomycetes infection. The patient continued anti-actinomycosis treatment for 1 month after surgery, and his condition stabilized after 8 months of follow-up.

**Conclusions:**

Imaging of pulmonary actinomycosis is easily confused with tuberculosis and tumors and is difficult to diagnose. Pulmonary actinomycosis should be considered in patients with a lung mass shadow and recurrent hemoptysis. When life-threatening hemoptysis complicates pulmonary actinomycosis, surgical resection may effectively control the condition and improve the prognosis.

## Introduction

Actinomycosis is a chronic suppurative, wasting, granulomatous infection caused by *Actinobacillus* spp. It predominantly involves the head, neck, and abdomen, with pulmonary involvement accounting for only 15%−20% of all cases (1). Although pulmonary actinomycosis is relatively rare, its true prevalence may be underestimated due to widespread misdiagnosis. Many cases initially diagnosed as refractory pneumonia, lung cancer, or tuberculosis may only be identified as actinomycosis after surgery or long-term follow-up. Owing to the lack of specificity in its clinical manifestations and imaging features, diagnostic delays often occur (2). Moreover, when combined with fatal hemoptysis, the disease progresses rapidly, and traditional anti-infective and interventional hemostatic methods are often challenged, requiring multidisciplinary collaboration to optimize diagnostic and treatment strategies.

Currently, the diagnosis of pulmonary actinomycosis relies on histopathology (e.g., detection of sulfur particles) and microbiological cultures; however, their low sensitivity and long culture cycles limit early confirmation of the diagnosis (3, 4). Furthermore, in patients with comorbid hemoptysis, conventional bronchial artery embolization may be ineffective due to the complexity of the blood supply to the lesion, making surgical resection a critical life-saving option. However, few systematic reports of such critical cases are available in the literature, and clinical decision-making lacks an evidence-based basis.

Herein, we report a complex case of a 74-year-old male patient who was finally diagnosed with pulmonary actinomycosis combined with fatal hemoptysis after 17 months of recurrent hemoptysis and multiple misdiagnoses and mistreatments. By reviewing the challenges of his diagnostic and treatment process, we aim to provide an evidence-based, comprehensive management experience for similar cases to improve clinical prognosis.

## Case presentation

**Figure 1:**
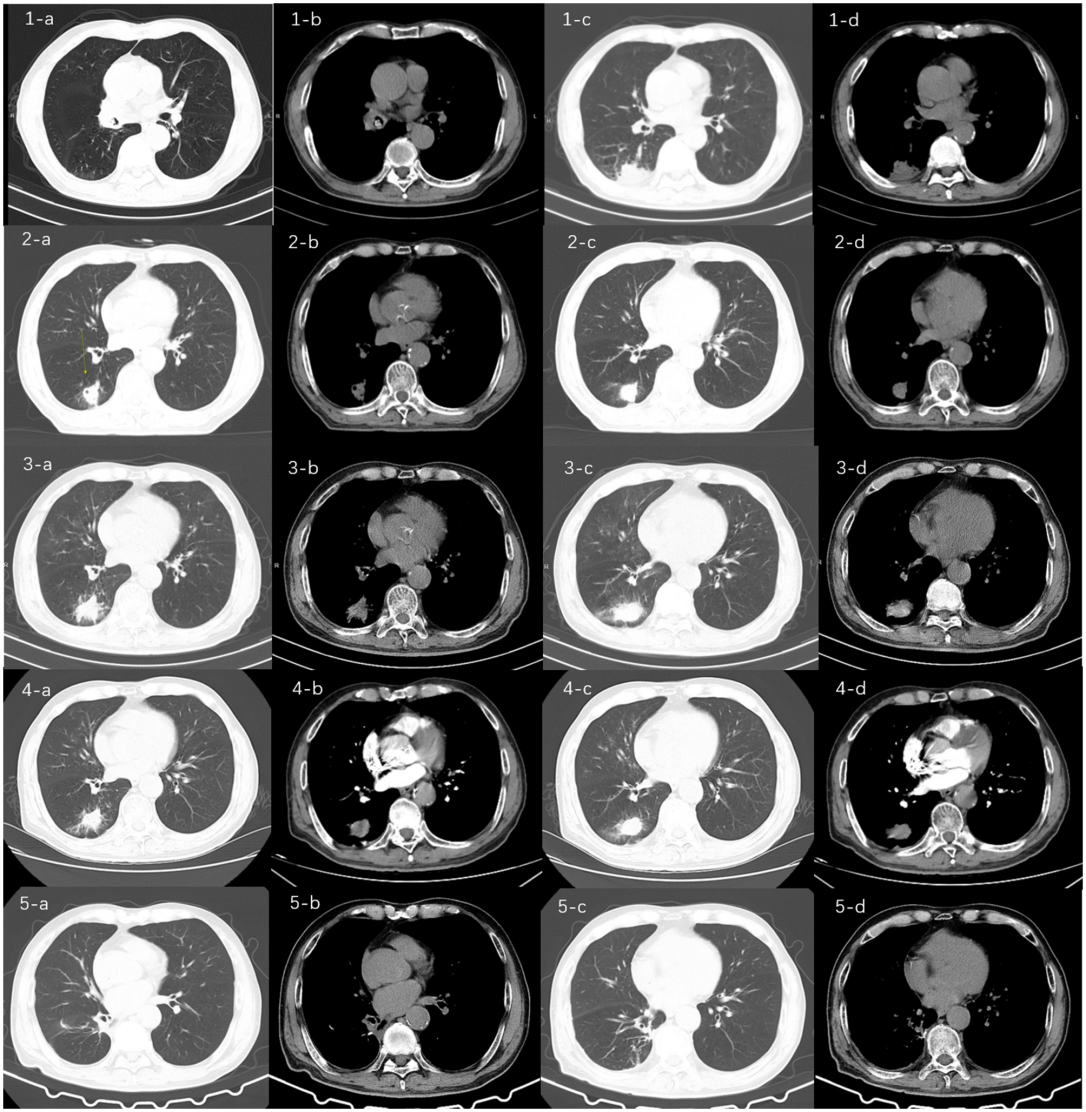
Chest computed tomography (CT) images of the patient over time. CT (**1-a, b, c, d**) at the time of initial admission (15 months prior) showed a dense shadow of the right middle bronchus, suggesting the possibility of a foreign body, along with partially solid infectious lesion in the right lower lobe. These findings prompted the first bronchoscopic intervention for foreign body removal; CT (**2-a, b, c, d**) from the second admission (11 months prior) showed a right lower lobe lesion with cavity formation, likely of infectious origin. The formation of a cavity is a characteristic progression of actinomycosis and was the primary reason for performing a CT-guided percutaneous lung biopsy to rule out malignancy or tuberculosis; CT (**3-a, b, c, d**) from the third admission (7 months prior) showed slight enlargement of the right lower lobe lesion and a reduction in cavity size. This mixed pattern of progression and regression under antibiotic therapy (moxifloxacin) raised diagnostic uncertainty, leading to the decision for bronchial artery embolization to manage life-threatening hemoptysis; and CT (**4-a, b, c, d**) from the fourth admission showed a lesion in the right lower lobe similar in appearance to the previous scan, with a further reduction in cavity size. The absence of significant bronchial artery dilation on CT angiography (not shown) and failure of medical hemostasis ultimately necessitated surgical resection, which provided the definitive diagnosis; CT(**5-a,b,c,d**) showed the patient's follow-up chest CT scan 2 months after surgery, which indicated no new lesions in the lungs and the patient's condition was stable.

**Figure 2:**
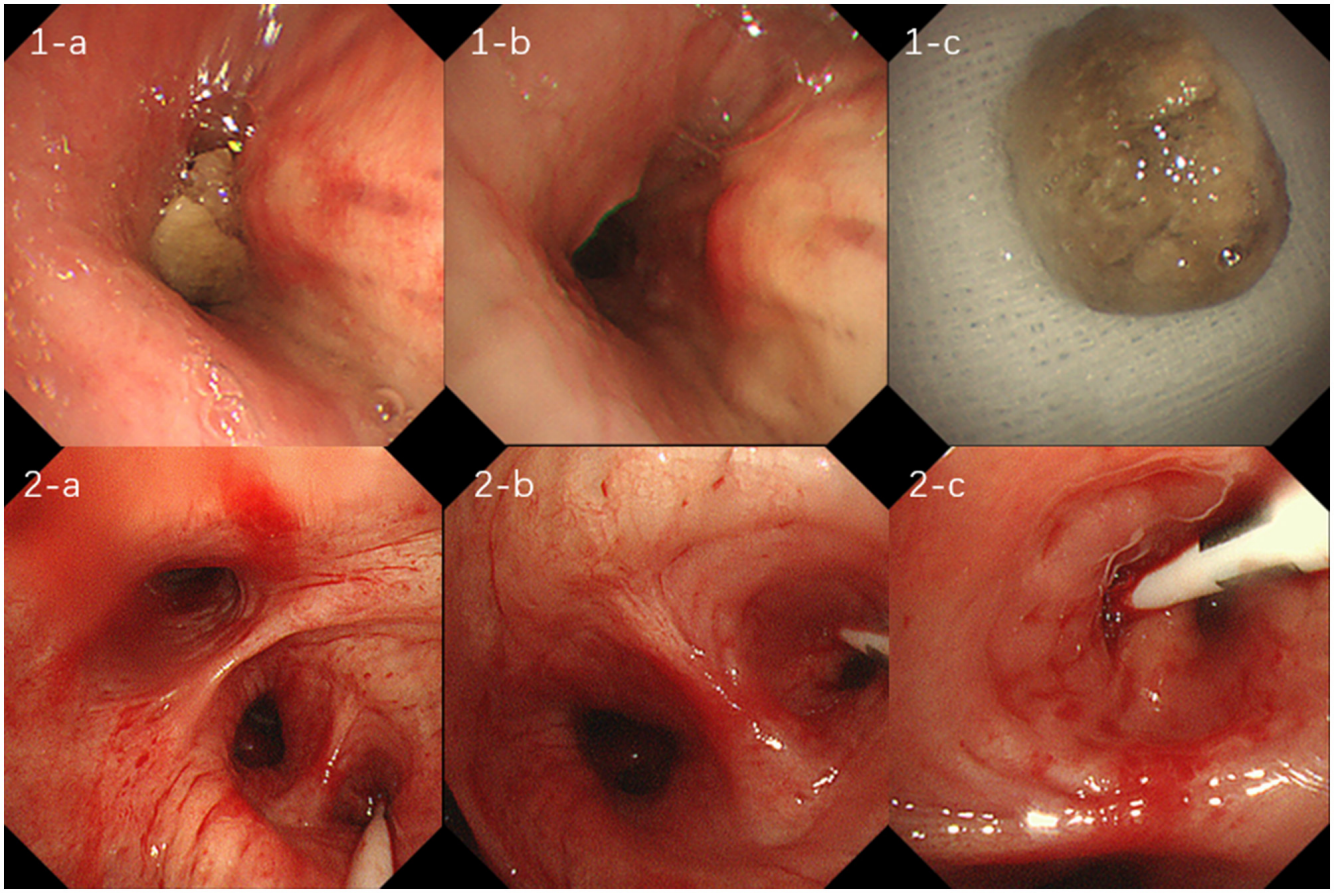
**(1-a, b, c)** show foreign body obstruction of the right middle bronchus, which was treated with transbronchoscopic removal of the foreign body; figures **(2-a, b, c)** suggest bronchial hemorrhage in the dorsal segment of the right lower lobe, treated with transbronchoscopic balloon occlusion to stop the hemorrhage.

**Figure 3:**
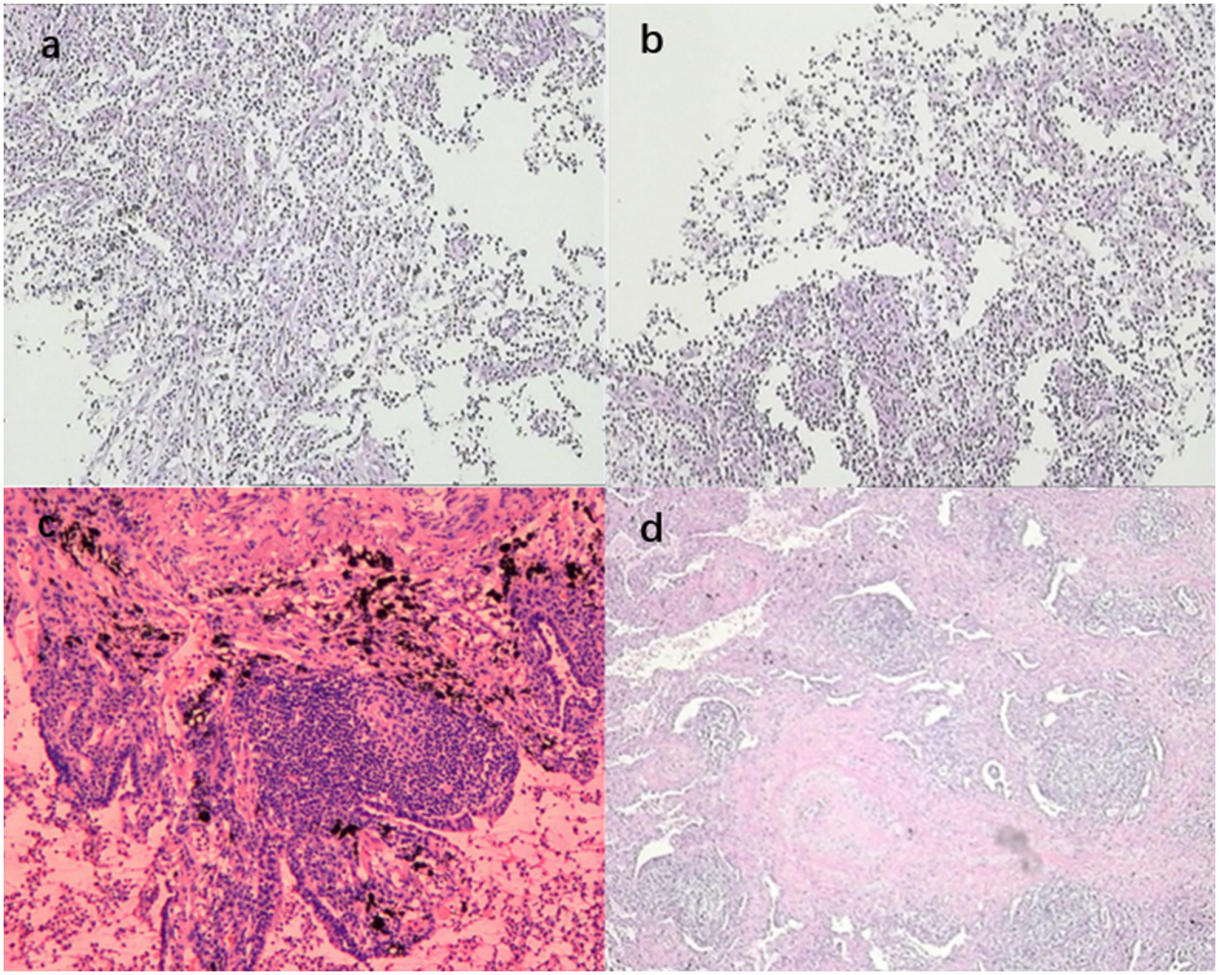
**(a, b)** show the biopsy pathology of the dorsal segment of the right lower lobe, revealing a small piece of lung tissue with extensive acute and chronic inflammatory cell infiltration; **(c, d)** show the post-operative pathology of the dorsal segment of the right lower lobe, revealing lung tissue with airway dilatation and pus accumulation, alveolar epithelial hyperplasia, interstitial fibrous tissue hyperplasia, extensive lymphocytic infiltration, and lymphoid follicle formation. The lesion was consistent with inflammatory granulomatous changes with regional bacterial clusters, which were suggestive of actinomycetes infection, and the periodic acid-Schiff staining was positive.

A 74-year-old male patient with a 9-year history of chronic obstructive pulmonary disease, whose symptoms were controlled by regular inhalation of fluticasone-salmeterol, and with a 10-year history of hypertension, whose blood pressure was controlled within normal range with regular medication, presented with the following: a long history of alcoholism, drinking two taels of white wine per meal. Fifteen months ago, he had intermittent hemoptysis for 2 months and went to the local hospital for a chest computed tomography (CT) scan, which showed a dense shadow of the right middle bronchus, with the possibility of a foreign body and partially solid infectious lesions being present in the lower lobe of the right lung (Figures 1:**1-a–d**). The patient was first admitted to our department, and the foreign body in the right middle bronchus was removed by bronchoscopy (Figures 2:**1-a–c**). He was discharged from the hospital with hemoptysis relieved by post-operative anti-infective treatment. Eleven months ago, the patient was re-admitted to our department with hemoptysis, and his CT showed a right lower-lobe lesion, with cavity formation, which was probably infectious (Figures 1:**2-a–d**). Further tests for tumor markers were normal. Additional tests were performed, including antinuclear antibody tests, extractable nuclear antigen tests, anti-neutrophil cytoplasmic antibody tests, T-cell spot test for tuberculosis infection, G test, galactomannan antigen detection test, cryptococcal antigen test, sputum bacterial culture, and tuberculosis culture, all of which were within normal limits. After reviewing the bronchoscopy, there were no obvious abnormalities, so a percutaneous lung aspiration biopsy with CT localization was performed on the right lower lung lesion, and the pathology suggested the occurrence of extensive acute and chronic inflammatory cell infiltration in the lung tissue (Figures 3a, b). The patient was discharged from the hospital with moxifloxacin anti-infective treatment. Since then, he continued to have intermittent low-volume hemoptysis, for which he did not consult a doctor, and he was admitted to our department for the third time 7 months ago due to an increase in hemoptysis volume (50 ml/day). CT showed that the right lower lung lesion was slightly enlarged and the cavity was reduced in size (Figures 1:**3-a–d**). After admission, he was administered posterior pituitary hormone, tranexamic acid, and thrombin to stop the hemoptysis, but the effect was unsatisfactory. Therefore, he underwent digital subtraction angiography-guided bronchial artery embolization and was discharged from the hospital with controlled hemoptysis after the operation.

The patient came to our emergency department for the fourth time on June 24, 2024, with an intermittent cough with hemoptysis, lasting for 17 months. On admission, the blood tests, ultrasensitive C-reactive protein (CRP), procalcitonin (PCT), coagulation function, and pro-brain natriuretic peptide levels were all within normal limits. Table 1 shows the first blood indices of the patient during his four visits to our hospital. As detailed in Table 1, the longitudinal trends in key biomarkers provided crucial insights into the patient's clinical course. For instance, the elevated D-dimer levels during episodes of active hemoptysis (e.g., the third admissions) were consistent with the breakdown of blood clots within the airways and the ongoing hemorrhagic event. Conversely, the normalization of D-dimer during quiescent periods reflected the stabilization of the hemorrhagic process. Similarly, fluctuations in inflammatory markers like CRP and PCT correlated with the intensity of the underlying infectious and inflammatory burden posed by the Actinomyces infection. The chest CT scan showed that the lesion in the lower lobe of the right lung was similar to the previous one, and the cavity in the lesion was reduced in size (Figures 1:**4-a–d**). The CT angiography did not suggest pulmonary embolism, and the bronchial arteries did not have any obvious tortuous dilatation. The patient was admitted to the respiratory ward under emergency hemostatic treatment with posterior pituitary hormone and tranexamic acid because of the high hemoptysis volume.

**Table 1:** Changes in laboratory indicators.

**Test**	**Admission 1**	**Admission 2**	**Admission 3**	**Admission 4**	**Reference range**
WBC	8.19	7.16	6.67	9.28	3.5–9.5 (10^9^/L)
PLT	186	153	139	130	125–350 (10^9^/L)
CRP	36.9	2.8	16.3	1.47	< 5 (mg/L)
PCT	0.088	0.04	–	0.009	< 0.1 (ng/ml)
PT	15.8	13.8	17.8	14.2	11.7–15.4 (s)
APTT	31.4	34.8	34.7	33.0	28–43.5 (s)
Fib	4.9	2.69	0.87	3.25	2–4 (g/L)
TT	17.8	17.5	24.0	18.7	< 21 (s)
D-dimer	0.58	0.38	>20	0.42	< 0.5 (μg/ml)
Cr	81	71	69	82	57–111 (μmol/L)
Na	143	143.5	143.4	145.3	137–147 (mmol/L)
Cl	105.8	104.8	107.6	102.3	99–110 (mmol/L)
K	3.12	3.88	3.52	3.74	3.5–5.3 (mmol/L)
Pro-BNP	249.3	–	–	75.5	5–125 (pg/ml)

WBC, white blood cell count; PLT, blood platelet; CRP, C-reactive protein; PCT, procalcitonin; PT, prothrombin time; APTT, activated partial thromboplastin time; Fib, fibrinogen; TT, thrombin time; Cr, creatinine; Na, serum sodium; Cl, serum chlorine; K, serum potassium; Pro-BNP, pro-brain natriuretic peptide.

In the respiratory ward, the patient continued to be treated with posterior pituitary hormone, hemagglutinin, and tranexamic acid for 3 days, and the hemoptysis was relieved. On the afternoon of the fourth day, the patient suffered multiple episodes of hemoptysis with no obvious triggers, and the volume of hemoptysis was approximately 150 ml, accompanied by a progressive decrease in oxygen saturation to 82%. The physical examination results were as follows: heart rate, 86 beats per minute; respiration, 24 beats per minute; blood pressure, 137/64 mmHg; and the patient was conscious and responsive. The patient presented with thick breath sounds in both lungs, with moist rales, together with a regular heart rhythm, soft abdomen, no edema in the limbs, and negative Babinski's sign on both sides. Hemoptysis and asphyxia were considered due to a right lower lung lesion. Considering the poor efficacy of pharmacological hemostasis, the patient was immediately sent to the bronchoscopy room for emergency rigid bronchoscopic balloon occlusion for hemostasis (Figures 2:**2-a–c**). After a multidisciplinary discussion between clinicians from interventional medicine, thoracic surgery, and the intensive care unit, it was considered that the patient's bronchial arteries did not have any obvious tortuous dilatation and that the efficacy of bronchial arterial embolism may be poor. Therefore, the team recommended a right lower lobectomy, which was performed during emergency thoracoscopy. The patient continued to receive symptomatic supportive treatment after surgery and had no hemoptysis. Post-operative pathology revealed airway dilatation and pus accumulation in the lung tissue, alveolar epithelial hyperplasia, interstitial fibrous tissue hyperplasia, lymphocytic infiltration, and lymphoid follicle formation. Additionally, the lesion was consistent with inflammatory granulomatous changes with regional bacterial clusters, which tended to be actinomycetes infection, and the periodic acid-Schiff staining was positive (Figures 3c, d). The patient's condition improved, so amoxicillin-clavulanate potassium 0.375 g was administered orally every 8 h, and the patient was discharged with medication.

**Figure 4:**
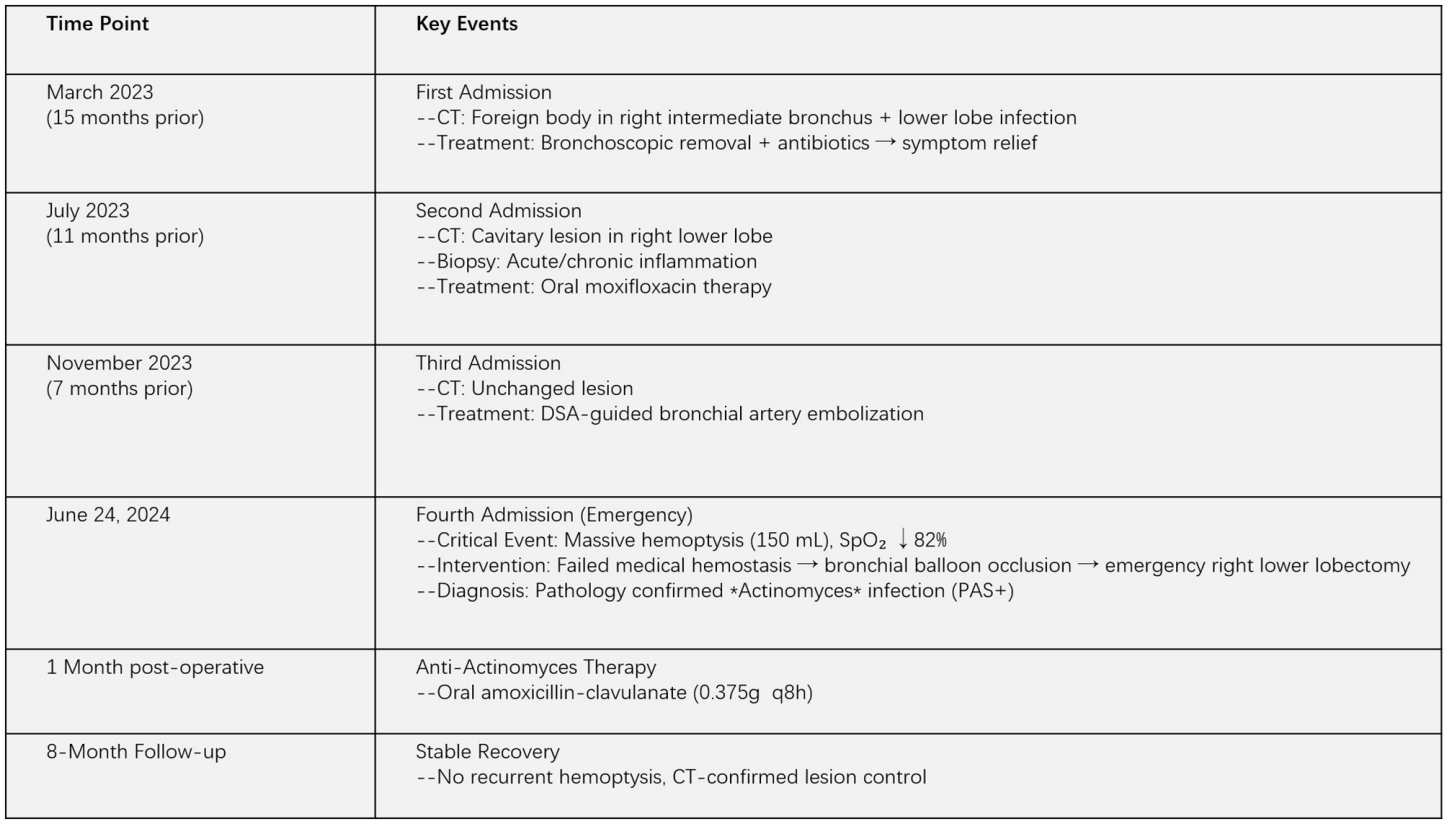
Hospitalization timeline and clinical treatments.

After discharge, the patient continued to receive anti-actinomycosis therapy at our respiratory medicine clinic for more than 1 month, after which the medication was discontinued. Two months later, the patient came to our clinic for a follow-up lung CT examination, and no new lesions were found (Figure 1:**5-a–d**). The timeline of the patient's entire treatment process is shown in Figure 4. Eight months after the operation, the patient was followed up again by telephone and reported a stable condition with no obvious discomfort.

## Discussion

The clinical presentation and imaging features of pulmonary actinomycosis often highly overlap with those of lung cancer, tuberculosis, or other infectious diseases, leading to significant diagnostic and therapeutic challenges (5, 6). The current case exemplifies the complexity of pulmonary actinomycosis and provides important insights into the clinical management of critically ill patients.

Actinomyces is a Gram-positive bacillus that may be found in the oral cavity, digestive tract, and genitourinary tract of healthy humans (7). *Actinomyces* is not normally pathogenic but can cause actinomycosis in individuals with reduced immune function. Pulmonary actinomycosis was first reported by Israel in 1878. It is considered one of the most difficult disease to differentiate and diagnose, with a high rate of misdiagnosis (8). Actinomycetes can be mistakenly inhaled through the oral cavity into the respiratory tract and invade the lungs, leading to pulmonary actinomycosis, which is the most common mode of infection (9). Poor oral hygiene, chronic alcohol abuse, and the inhalation of foreign objects are common risk factors (10). In this case, the patient had a history of chronic alcohol abuse, and the lung CT at the initial visit suggested a foreign body in the right middle lobe bronchus. Therefore, it was considered that actinomycetes were inhaled with the foreign body into the respiratory tract causing pulmonary infection, which aligned with the commonly reported risk factors.

The clinical manifestations of pulmonary actinomycosis are varied, with common symptoms including fever, cough, hemoptysis, and chest pain, similar to those of common respiratory diseases. When the patient coughs up yellow sulfurous particulate matter, it is diagnostic of the disease; however, this is clinically rare (11). The current patient presented with recurrent hemoptysis, possibly due to the proteolytic enzymes released by actinomycetes. These enzymes form a lytic, purulent inflammation around the lung tissues, which destroys the blood vessel walls, causing hemorrhage (12). Therefore, in lung infections with recurrent hemoptysis, it is necessary to maintain vigilance for *Actinomyces* infection. However, since hemoptysis is also often seen in other lung diseases, such as tuberculosis and lung cancer, it is difficult to confirm the diagnosis based on clinical symptoms alone.

CT imaging of pulmonary actinomycetes lacks specificity and can easily be misdiagnosed as malignancy, tuberculosis, or other infections (13). As aspiration of foreign material is the most common mechanism of function, most lesions are located in the lower lobes. In the early stage, most lesions appear as subpleural patchy shadows, and in the middle and late stages, the lesions appear as clustered shadows. In typical cases, the central cavity is seen with fluid and small rounded gaseous shadows. The gas and liquefied tissues do not form a gas–liquid plane, but are instead suspended in the necrotic foci, which is the most characteristic feature of pulmonary actinomycosis that distinguishes it from other cavitary lesions (5). In our patient, the site of onset was the right lower lung near the pleura, which was initially a mass shadow and appeared as the lesion progressed to cavitation, consistent with the development of pulmonary actinomycosis. Using other imaging modalities, such as positron emission tomography/CT, pulmonary actinomycosis can also show a high degree of uptake of the lesion, so it does not have a definite value in the differential diagnosis with malignancy (14).

The pathophysiology of massive hemoptysis in pulmonary actinomycosis is distinct and stems from the organism's unique pathogenic process. Actinomyces species induce a chronic suppurative and granulomatous inflammation, characterized by the formation of sulfur granules—microcolonies of bacteria embedded in a matrix of calcium phosphate and host proteins. This inflammatory response is highly destructive and has a marked tendency to extend across anatomical boundaries, including fissures and the pleural cavity, a feature less commonly seen in tuberculosis or carcinoma. The proteolytic enzymes released by both the bacteria and the host's inflammatory cells lead to progressive tissue necrosis, liquefaction, and cavitation. Crucially, this lytic process erodes the walls of adjacent pulmonary and bronchial vessels, resulting in hemorrhage. Furthermore, the accompanying intense fibrotic reaction can distort vascular architecture and impair healing, creating a nidus for recurrent bleeding. This explains why bronchial artery embolization, which targets hypertrophied systemic arteries, may fail in actinomycosis if the bleeding source is primarily from pulmonary arteries or due to diffuse inflammatory neovascularization within the lesion, ultimately necessitating surgical resection for definitive control as in the present case.

The diagnosis of pulmonary actinomycosis is often challenging, and its confirmation relies on microbiological or pathological evidence (15). This is because the detection of actinomycetes in sputum or bronchoalveolar lavage fluid may represent colonization, and cultures are often negative due to their anaerobic nature, prior antibiotic therapy, and inadequate culture techniques (1). Some cases of pulmonary actinomycosis have been diagnosed due to an initial diagnosis of malignancy and subsequent surgical procedures, with the diagnosis ultimately confirmed by surgical pathology (16, 17). CT-guided lung puncture biopsy is a safer diagnostic technique, but a single biopsy does not always lead to a definitive diagnosis, and multiple multi-site biopsies should be performed to increase the positivity rate (18). Meanwhile, histopathology combined with tissue microbiological culture can further improve the clinical diagnosis rate. The diagnostic value of bronchoscopy was previously considered to be low, but with the advancement of testing technology, an increasing number of cases can now be diagnosed clearly by bronchoscopy (19, 20). In the present case, the diagnosis was not clear by bronchoscopy and CT-guided lung puncture biopsy, and was finally clarified by surgical pathology. This case also demonstrates that both clinicians and laboratory technicians lack sufficient knowledge about this disease.

The formidable diagnostic challenge of pulmonary actinomycosis primarily stems from its “great imitator” nature, which leads to frequent confusion with lung malignancy or tuberculosis. Clinically, symptoms such as hemoptysis, weight loss, and night sweats are non-specific and can be present in all three conditions. Radiologically, the mass-like appearance, cavitation, and pleural involvement observed in our patient are features highly reminiscent of lung cancer or tuberculous cavities. Microbiologically, the fastidious growth requirements of actinomyces and the common prior use of antibiotics result in low culture positivity. In the present case, the initial foreign body obscured the diagnosis, and subsequent CT-guided biopsy only revealed inflammation, highlighting that a single negative biopsy cannot reliably exclude the disease. This diagnostic dilemma often leads to a protracted course, as seen in our patient's 17-month history, during which he underwent multiple invasive procedures including bronchial artery embolization before a definitive diagnosis was achieved surgically.

The initial treatment of choice for pulmonary actinomycosis includes a high-dose, long-course intravenous infusion of penicillin (18–24 million units/d) for 2–6 weeks, followed by amoxicillin for 6–12 months (3, 12). Other drugs, such as sulfonamides and tetracyclines, as well as erythromycin and moxifloxacin, are alternative treatment options (3). The prognosis is usually good, and no surgical treatment is required. However, surgery may be required when drug therapy is unsatisfactory. The principle of surgery is to resect the diseased lung and treat the complications. In this case, the patient was given moxifloxacin after several visits to the clinic. However, due to the failure to identify the causative organism, no long-term adherence to the use of the drug was observed. Thus, the treatment efficacy was poor. The decision to proceed with surgical resection in this case was reached through a multidisciplinary consensus, highlighting its critical role in managing life-threatening complications of pulmonary actinomycosis. For this patient with massive hemoptysis unresponsive to medical and initial interventional therapy, and given the angiographic absence of significant bronchial artery dilatation suggesting a potentially poor response to repeat embolization, emergency surgery became the definitive life-saving measure. The primary advantage of surgery in this context was twofold: it provided immediate and definitive control of the hemorrhagic source, and it supplied ample tissue for histopathological examination, which was ultimately diagnostic. This underscores that in critical scenarios, surgery transcends its therapeutic role and serves as the ultimate diagnostic tool. After surgery, the patient continued to take amoxicillin-clavulanate potassium for more than 1 month, and his condition was stable at 8 months of follow-up.

In this case, the patient received oral amoxicillin-clavulanate potassium for only 1 month following surgical resection. This decision was based on several specific considerations. First, the source of persistent infection—the right lower lobe—was completely removed via lobectomy, achieving a definitive anatomical cure. Second, the post-operative pathology confirmed that the inflammatory granulomatous changes were localized, with clear surgical margins, suggesting a low risk of residual disseminated disease. Finally, given the patient's advanced age and potential for long-term antibiotic-related adverse effects, a shorter course was deemed a reasonable compromise under close clinical surveillance. The patient's stability at the 8-month follow-up supports the adequacy of this individualized approach. Nevertheless, the standard long-course antibiotic regimen remains the cornerstone of treatment, especially when surgery is not performed or when disease is extensive.

## Conclusion

The imaging manifestations of pulmonary actinomycosis closely overlap with those of lung tumors and tuberculosis, which can lead to clinical misdiagnosis. Therefore, it is important to be vigilant for this disease in cases of recurrent hemoptysis with lung mass shadow, and the definitive diagnosis relies on histopathological or microbiological evidence. Moreover, surgical intervention can significantly improve the prognosis of critically ill patients with life-threatening hemoptysis.

## Data Availability

The original contributions presented in the study are included in the article/supplementary material, further inquiries can be directed to the corresponding author.
